# Rapamycin Ameliorates Defects in Mitochondrial Fission and Mitophagy in Glioblastoma Cells

**DOI:** 10.3390/ijms22105379

**Published:** 2021-05-20

**Authors:** Paola Lenzi, Rosangela Ferese, Francesca Biagioni, Federica Fulceri, Carla L. Busceti, Alessandra Falleni, Stefano Gambardella, Alessandro Frati, Francesco Fornai

**Affiliations:** 1 Department of Translational Research and New Technologies in Medicine and Surgery, University of Pisa, Via Roma 55, 56126 Pisa, Italy; paola.lenzi@unipi.it; 2 I.R.C.C.S. Neuromed, Via Atinense 18, 86077 Pozzilli, Italy; ferese.rosangela@gmail.com (R.F.); francesca.biagioni@neuromed.it (F.B.); carla.busceti@neuromed.it (C.L.B.); stefano.gambardella@neuromed.it (S.G.); alessandro.frati@uniroma1.it (A.F.); 3 Department of Clinical and Experimental Medicine University of Pisa, via Roma 55, 56126 Pisa, Italy; federica.fulceri@unipi.it (F.F.); alessandra.falleni@unipi.it (A.F.); 4 Department of Biomolecular Sciences, University of Urbino “Carlo Bo”, Piazza S. Andrea 34, 61029 Urbino (PU), Italy; 5 Neurosurgery Division, Human Neurosciences Department, Sapienza University, 00135 Roma, Italy

**Keywords:** OPA1, FIS1, DRP1, VPS34, ULK1, AMBRA1, PINK1, PARKIN, autophagy, mitochondria

## Abstract

Glioblastoma (GBM) cells feature mitochondrial alterations, which are documented and quantified in the present study, by using ultrastructural morphometry. Mitochondrial impairment, which roughly occurs in half of the organelles, is shown to be related to mTOR overexpression and autophagy suppression. The novelty of the present study consists of detailing an mTOR-dependent mitophagy occlusion, along with suppression of mitochondrial fission. These phenomena contribute to explain the increase in altered mitochondria reported here. Administration of the mTOR inhibitor rapamycin rescues mitochondrial alterations. In detail, rapamycin induces the expression of genes promoting mitophagy (*PINK1*, *PARKIN*, *ULK1*, *AMBRA1*) and mitochondrial fission (*FIS1*, *DRP1*). This occurs along with over-expression of *VPS34*, an early gene placed upstream in the autophagy pathway. The topographic stoichiometry of proteins coded by these genes within mitochondria indicates that, a remarkable polarization of proteins involved in fission and mitophagy within mitochondria including LC3 takes place. Co-localization of these proteins within mitochondria, persists for weeks following rapamycin, which produces long-lasting mitochondrial plasticity. Thus, rapamycin restores mitochondrial status in GBM cells. These findings add novel evidence about mitochondria and GBM, while fostering a novel therapeutic approach to restore healthy mitochondria through mTOR inhibition.

## 1. Introduction

In glioblastoma multiforme (GBM), mTOR is activated and autophagy is inhibited [1]. This, in turn, inhibits cell differentiation and fosters proliferation of glioblastoma stem progenitor cells [2]. Autophagy suppression is documented by a decrease in LC3B-2 and beclin1, which is documented at gold-standard transmission electron microscopy (TEM) [3]. A chain of events exists, where suppressed autophagy fosters tumor progression, relapse, and radio-resistance [4]. When autophagy is restored, GBM cells differentiate [5,6]. Upregulation of mTORC1 determines autophagy suppression, which stimulate GBM cancer stem cells, as documented in glioblastoma cell lines, primary GBM cells cultures, and implanted GBM xenograft [5,6,7,8,9,10]. It is likely that a beneficial role of autophagy depends on the clearance of specific proteins, which sustain tumor progression, such as the prion protein PrPc, which promotes the growth of GBM [7,11]. A fascinating hypothesis suggests that other autophagy-dependent effects, including mitochondrial turnover, may be relevant in the natural course of GBM [11,12,13,14,15,16]. Thus, mitochondrial alterations and protein misfolding co-exist in GBM [13,14,15,16,17,18,19]. In fact, in a very recent study we documented a suppression of mitochondrial biogenesis, which was restored by rapamycin [10]. To encompass the knowledge of mitochondrial status in GBM cells in the present study we document mitochondrial fission, fusion, and mitophagy. The study was carried out in baseline conditions and at various time intervals following a short challenge with rapamycin. A quantitative analysis is carried out of both gene and protein expression by morphometric stoichiometry at mitochondrial level. Mitochondrial fission, and mitophagy were related to mitochondrial status. In fact, fission and mitophagy are expected to buffer cell damage generated by altered mitochondria [20,21,22]. This study wishes to add evidence on how specific alterations of mitophagy and mitochondrial status may be relevant for GBM malignancy [13,14,16,23,24,25,26,27,28,29,30,31,32,33].

## 2. Results

### 2.1. Dose–Response and Time-Course of Rapamycin-Induced Mitochondrial Number and Alterations

These experiments were carried out by using various doses (1 nM, 10 nM, and 100 nM) and times (12 h, 24 h, and 72 h) of rapamycin continuous exposure in order to assess which dose and time were most appropriate to assess the effects produced at various time intervals of rapamycin withdrawal. Thus, these pilot experiments were designed to select the dose and time of a short exposure to rapamycin, which were effective to modify persistently the mitochondrial status. As shown in Figure S1, 24 h exposure to 10 nM rapamycin produces the highest number of healthy and functional mitochondria assessed with MitoTracker-Red (representative pictures of Figure S1A and graphs of Figure S1B) as well as the maximal number of total mitochondria and the lowest number of altered mitochondria counted at ultrastructural morphometry (Figures S2–S5). In fact, at 24 h of continuous 10 nM rapamycin exposure mitochondria were in excess of 150% of controls. This dosing and timing reproduce previous published data, when the dose–response of rapamycin to stimulate mitochondrial biogenesis reached a plateau at 10 nM rapamycin [10]. These experimental conditions were kept steady all over the study in order to measure changes taking a place at prolonged time intervals (up to 21 d) of rapamycin withdrawal. This allowed to check the persistency of improved mitochondrial status produced by the long-lasting effects produced by rapamycin, even when rapamycin was no longer present.

### 2.2. Long-Lasting Increase of Mitochondria and Clearance of Altered Mitochondria

**Figure 1:**
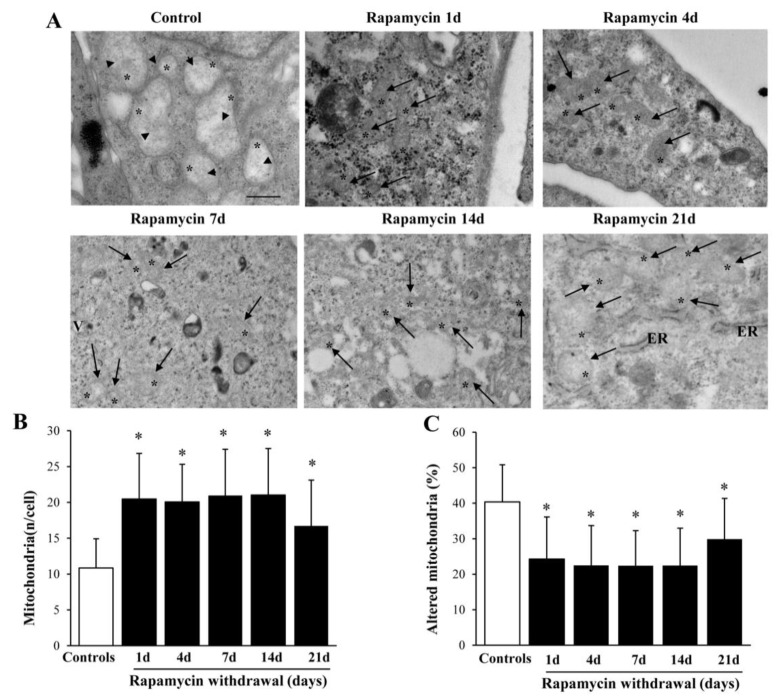
Rapamycin increases the number of mitochondria and reduces altered mitochondria time-dependently. (**A**) Representative pictures of mitochondria from controls and following different time of 10 nM rapamycin withdrawal. Both normally structured, small (arrows), and altered, large mitochondria (arrowhead) are shown in these pictures. Graph (**B**) reports the total number of mitochondria per cell. Graph (**C**) reports the percentage of altered mitochondria. Counts represent the mean ± SD from N = 50 cells per group. Asterisk (*) = mitochondria; ER = endoplasmic reticulum. * *p* ≤ 0.05 compared with control. Scale bars = 1 μm.

Following a short-time rapamycin exposure an increase of mitochondria was detected during rapamycin withdrawal. As shown in representative pictures, Figure 1A mitochondria observed in controls become denser and smaller following rapamycin 10 nM. The increase in total mitochondria was remarkably steady from 1 d up to 21 d following a rapamycin challenge (10 nM, for 24 h graph of Figure 1B), and it was concomitant with a long-lasting decrease in the number of altered mitochondria (graph of Figure 1C). The increase in total mitochondria also depend on newly synthesized mitochondria on mitochondrial biogenesis reported in Ferese et al. [10], which exceeds the decrease in the amount of altered organelles reported here. Again, an increased mitochondrial fission may contribute to this effect; therefore, such an issue will be specifically addressed later in the study. It is remarkable the long persistency of the effect which does not vanish even keeping the cells without rapamycin for 21 d. This calls for a rapamycin-induced plasticity in determining mitochondrial status.

### 2.3. Rapamycin Increases the Histofluorescence from Healthy Mitochondria

**Figure 2:**
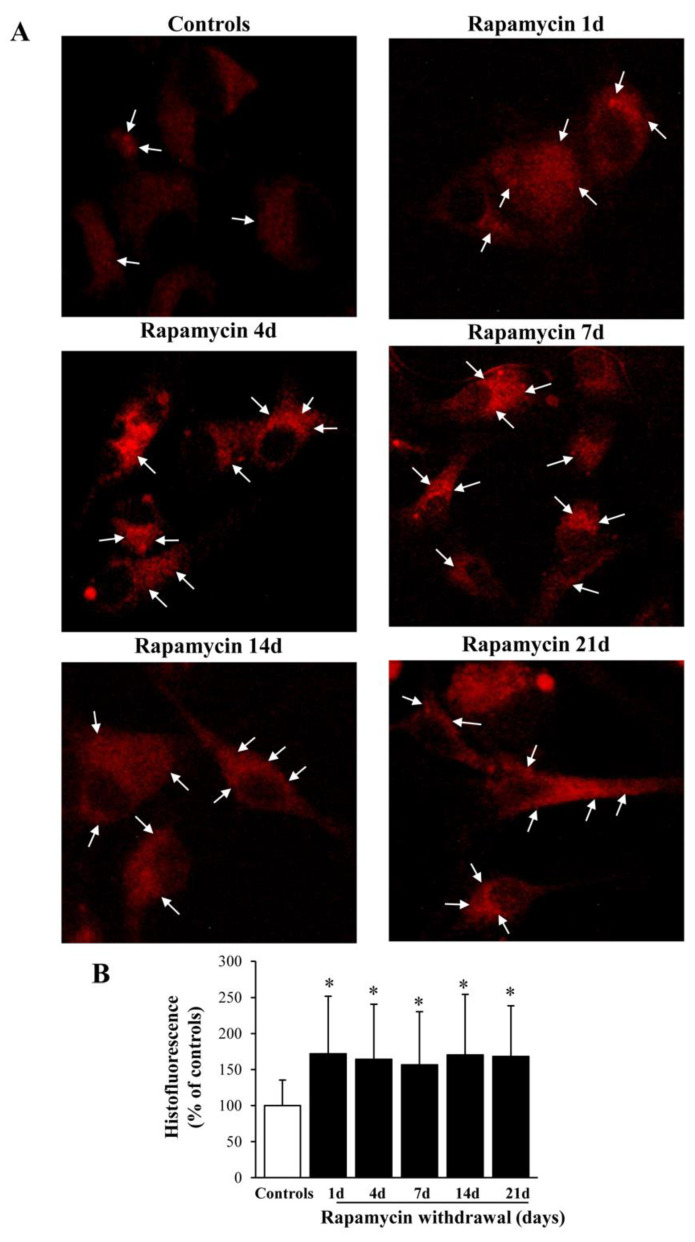
During rapamycin withdrawal a persistent increase in the amount of healthy mitochondria takes place. (**A**) Representative pictures stained with MTR showing healthy mitochondria (arrows) from controls or rapamycin (10 nM for 24 h) at various time intervals following withdrawal (from 1 d up to 21 d). It is evident how rapamycin treatment produces increased fluorescence, which is steady at various time intervals. This is counted in the graph (**B**), which reports the percentage of MTR histofluorescence compared with control, which persists steadily elevated up to 21 d. Counts represent the mean ± SD from N = 50 cells per group. ∗ *p* ≤ 0.05 compared with control. Scale Bar = 20 μm.

As expected, representative pictures of Figure 2 show a clear and steady increase in the fluorescent signal produced by the dye Mito-Tracker Red, which stains functional mitochondria [34]. Rapamycin–induced improvement of mitochondrial status persists up to 21 d following withdrawal (Figure 2A,B). This may depend on the transcription of specific genes.

### 2.4. Rapamycin Increases Mitochondrial Fission Genes FIS1 and DRP1, While Decreases Fusion Gene OPA1

**Figure 3:**
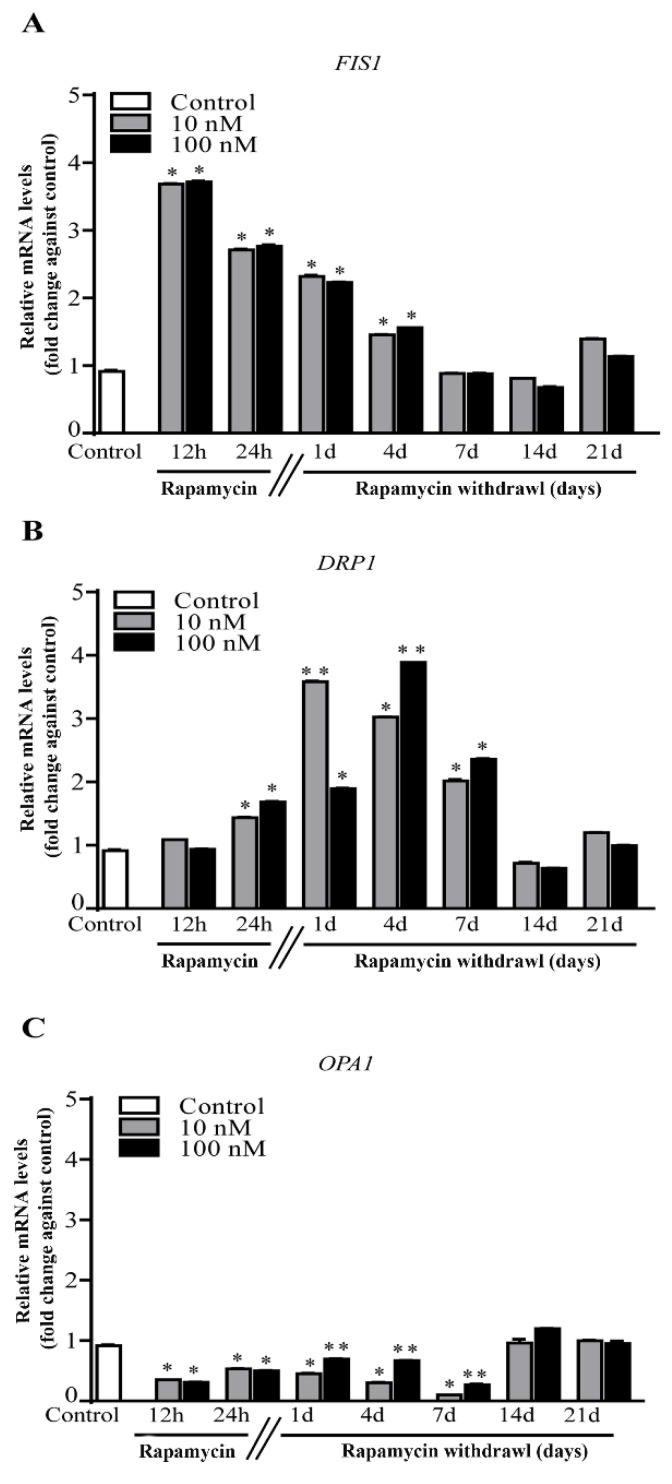
Effects of rapamycin on fission and fusion genes. RT-PCR for (**A**) *FIS1*, (**B**) *DRP1*, (**C**) *OPA1*. The fission-related genes *FIS1* and *DRP1* were both increased by rapamycin, with an earlier increase concerning *FIS1*, which reached back control levels at 7 days. *DRP1* increased later reaching back control levels at 14 days. The fusion antigen *OPA1* decreased following both a low and high dose of rapamycin and ruse back to control levels at 14 days. Counts represent the mean ± SD. * *p* ≤ 0.05 compared with control. ** *p* ≤ 0.05 compared with control and 10 nM rapamycin.

As expected, rapamycin alters expression of genes related to mitochondrial dynamics. In fact, rapamycin increases the expression of genes related to mitochondrial fission (*FIS1* and *DRP1*, Figure 3A,B, respectively), while it decreases the gene *OPA1* related to mitochondrial fusion. This is expected to contribute to the increase in mitochondrial number.

### 2.5. Rapamycin Differently Alters the Autophagy Genes VPS34, ULK1 and AMBRA1

**Figure 4:**
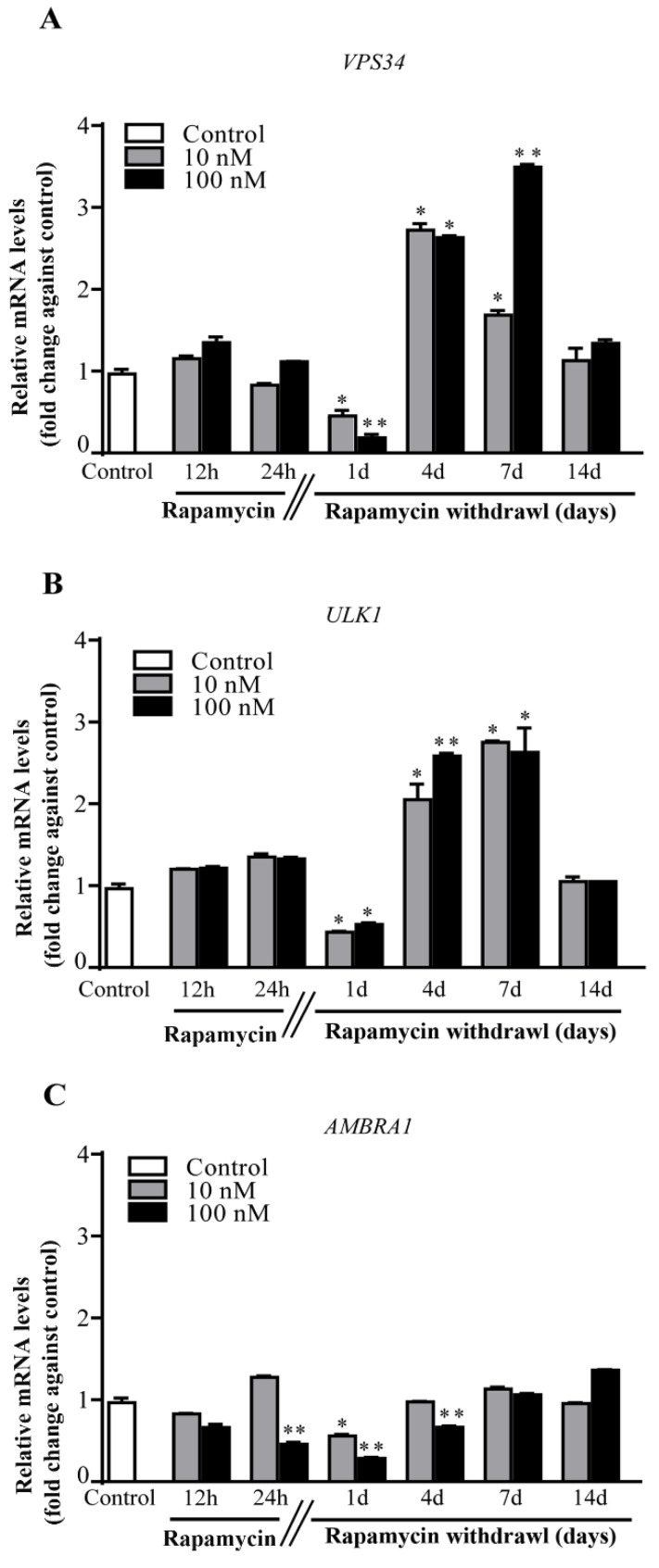
Rapamycin increases the mitophagy genes *VPS34* and *ULK1* while decreases *AMBRA1*. RT-PCR for (**A**) *VPS34*, (**B**) *ULK1*, (**C**) *AMBRA1*. In detail, *VPS34* and *ULK1* were depressed compared with controls a 1 d following rapamycin (both 10 nM and 100 nM) withdrawal. Later on, a marked increase in both genes was measured at 4 d and 7 d, going back to control levels at 14 d. In contrast, *AMBRA1* never increased compared with controls. In detail, as occurring for *VPS34* and *ULK1*, *AMBRA1* decreases at 1 d following rapamycin (both 10 nM and 100 nM) withdrawal. Such a decrease was evident also during rapamycin administration and at 4 d of withdrawal, when the highest dose (100 nM) of rapamycin was administered. Counts represent the mean ± SD. * *p* ≤ 0.05 compared with control. ** *p* ≤ 0.05 compared with control and 10 nM rapamycin.

Rapamycin persistently increases (for 7 d of rapamycin withdrawal) in excess of threefold the *VPS34* gene, which is upstream in the autophagy cascade (Figure 4A). This overlaps with the specific mitophagy gene *ULK1* (Figure 4B). It is remarkable that, both genes being overexpressed, undergo a short transient inhibition at 1 d of rapamycin withdrawal (Figure 4A,B). Contrary wise, the levels of the mitophagy gene *AMBRA1*, were different at 10 nM and 100 nM of rapamycin (Figure 4C). In fact, at 10 nM rapamycin *AMBRA1* was not significantly affected, whereas at the highest dose (100 nM) rapamycin, significantly decreased *AMBRA1* compared with control. This might be due to the fact that *AMBRA1*, plays a complex role in mitophagy since depending on the amount of phosphorylation it might up- or down-regulate *ULK1*, thus increasing or suppressing mitophagy [35,36].

### 2.6. Rapamycin Withdrawal Modulates PINK1 and PARKIN Genes

**Figure 5:**
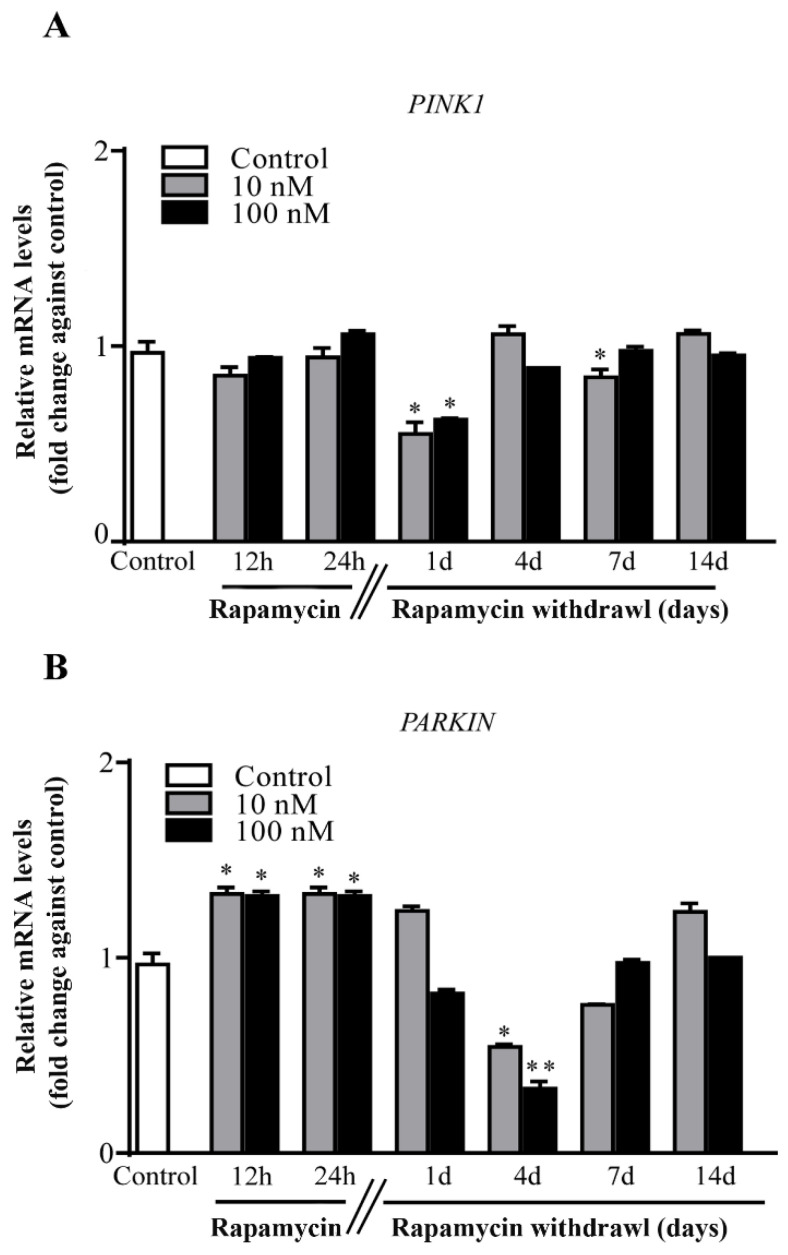
Rapamycin transiently modifies expression of *PINK1* and *PARKIN*. RT-PCR for (**A**) *PINK1* and (**B**) *PARKIN*. *PINK1* never increases following rapamycin. This gene undergoes a decrease following 1 d of rapamycin (10 nM and 100 nM withdrawal), with a slight decrease also measured at 7 d following the lowest dose. In contrast, *PARKIN* rose early on during rapamycin (both doses) administration and similarly to *PINK1* decreased transiently at 4 d following both doses of rapamycin. Counts represent the mean ± SD. * *p* ≤ 0.05 compared with control. ** *p* ≤ 0.05 compared with control and 10 nM rapamycin.

Despite decreased level (*PINK1* Figure 5A) or increased amount (*PARKIN* Figure 5B) were detected during, or short after rapamycin exposure, a marked decrease in *PARKIN* expression was observed at 4 d during rapamycin withdrawal (Figure 5B). This effect markedly contrasts with the specific over expression of mitophagy-related genes and suggests a different regulation in the expression of genes related to mitochondrial removal (consider here also the discrepancy described for *AMBRA1* compared with *ULK1*). Thus, increased expression of *PARKIN* seems to be required for a short time in order to trigger the mitophagy process. Similarly, the amount of *PINK1* changes transiently following rapamycin (Figure 5A).

### 2.7. Rapamycin Polarizes Increased LC3 within Mitochondria

**Figure 6:**
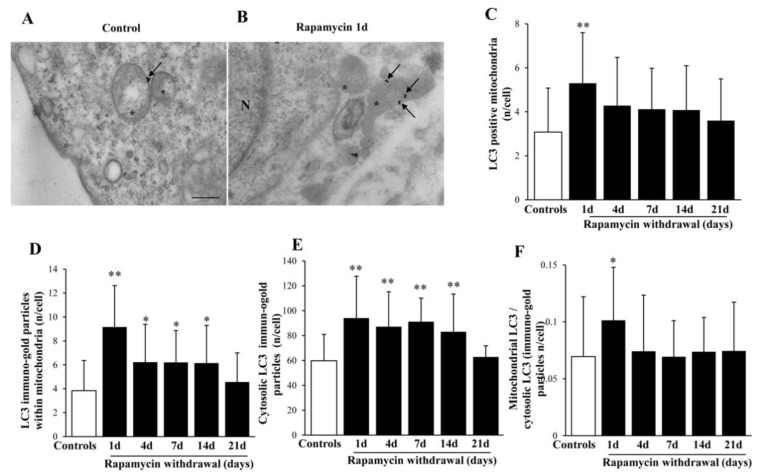
Rapamycin increases the amount of LC3-positive mitochondria. Representative TEM micrograph showing LC3-positive mitochondria from control (**A**) and following 1 d rapamycin withdrawal (**B**). Arrows point to LC3 immuno-gold particles within mitochondria. Graphs report the LC3-positive mitochondria (**C**), LC3 immuno-gold particles within mitochondria (**D**), the cytosolic amount of LC3 (**E**), and the ratio of mitochondrial to cytosolic LC3 particles (**F**). Counts represent the mean ± SD from N = 50 cells per group. * *p* ≤ 0.05 compared with control. ** *p* ≤ 0.05 compared with controls and 21 d. Scale Bar = 0.5 μm, Asterisk (*) = mitochondria, N = nucleus.

Despite being roughly increased in the cell, a specific compartmentalization of LC3 following rapamycin was measured at mitochondrial level (Figure 6). This is quite consistent with the persistency of rapamycin-induced increase in autophagy as evidenced by VPS34 up to 14 d (graph Figure S6A). Nonetheless, while LC3 possesses a mitochondrial polarization, VPS34 does not show such a preferential topography (graphs of Figure S6B–D), which suggests a rather generalized and upstream signal for autophagy for VPS34 compared with LC3 which best targets specific compartment to be digested [3].

It is well known that rapamycin increases LC3 in the cell and within ATG vacuoles. The present study adds evidence for a powerful compartmentalization of LC3 towards mitochondria exerted by rapamycin. As shown in representative Figure 6A,B, at 1 d following rapamycin 10 nM withdrawal, LC3 is evident at the mitochondrial level. In detail, the amount of mitochondria staining with LC3 increases almost twofold of control (Figure 6B,C). Such a finding is more evident when counting the number of LC3 particles within mitochondria (Figure 6D). This is compatible with the classic increase of cytosolic LC3 induced by rapamycin and confirmed here in Figure 6E. Nonetheless when plotting the increase of LC3 particles within mitochondria vs. cytosolic LC3, the effects of rapamycin was more powerful to address LC3 within mitochondria rather than towards stochastic cytosolic areas (Figure 6F). In these graphs, another phenomenon is apparent, which concerns the persistency of high LC3 levels, which vanishes at 14 d both within mitochondria and cytosol. The graph shows a significant compartmentalization of LC3 at mitochondrial level, such a placement of LC3 particles counted at immuno-gold stoichiometry is comparable to the compartmentalization of LC3 within ATG vacuoles. This makes mitochondria as a key site for LC3 and confirms the concerted pattern of mitophagy in the autophagy process [10].

### 2.8. Rapamycin Increases Mitophagy-Related Proteins within Mitochondria

**Figure 7:**
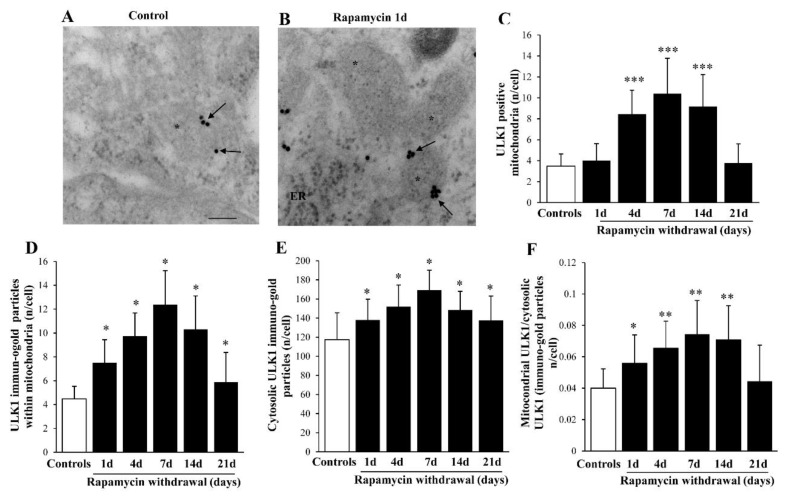
Representative immuno-gold and polarization graphs for the mitophagy marker ULK1. Representative TEM micrograph showing ULK1-positive mitochondria from control (**A**) and following 1 d rapamycin withdrawal (**B**). Arrows point to ULK1 immuno-gold particles within mitochondria. Graphs report the ULK1-positive mitochondria (**C**), ULK1 immuno-gold particles within mitochondria (**D**), the cytosolic amount of ULK1 (**E**), and the ratio of mitochondrial to cytosolic ULK1 particles (**F**). Values reported in the graph correspond to the mean ± SD. Mitochondria were counted in 50 cells per group. * *p* ≤ 0.05 compared with controls. ** *p* ≤ 0.05 compared with controls and 21 d. *** *p* ≤ 0.05 compared with controls, 1 d and 21 d. Scale Bar = 0.2 μm, Asterisk (*) = mitochondria, ER = endoplasmic reticulum.

**Figure 8:**
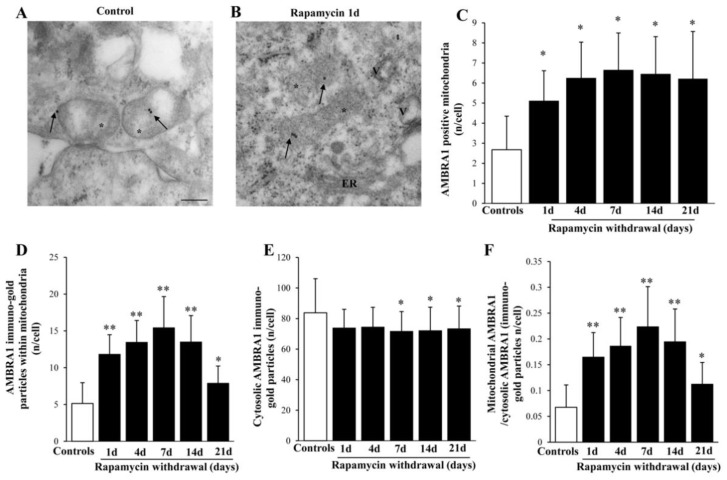
Representative immuno-gold and polarization graphs for the mitophagy marker AMBRA1. Representative TEM micrograph showing AMBRA1-positive mitochondria from control (**A**) and following 1 d rapamycin withdrawal (**B**). Arrows point to AMBRA1 immuno-gold particles within mitochondria. Graphs report the AMBRA1-positive mitochondria (**C**), AMBRA1 immuno-gold particles within mitochondria (**D**), the cytosolic amount of AMBRA1 (**E**), and the ratio of mitochondrial to cytosolic AMBRA1 particles (**F**). Values reported in the graph correspond to the mean ± SD. Mitochondria were counted in 50 cells per group. * *p* ≤ 0.05 compared with controls. ** *p* ≤ 0.05 compared with controls and 21 d. Scale Bar = 0.5 μm, Asterisk (*) = mitochondria, ER = endoplasmic reticulum.

**Figure 9:**
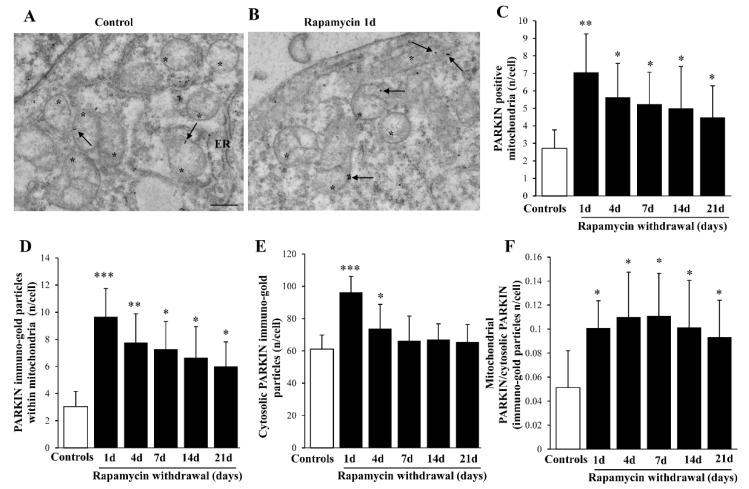
Representative immuno-gold and polarization graphs for PARKIN. Representative TEM micrograph showing PARKIN-positive mitochondria from control (**A**) and following 1 d rapamycin withdrawal (**B**). Arrows point to PARKIN immuno-gold particles within mitochondria. Graphs report the PARKIN-positive mitochondria (**C**), PARKIN immuno-gold particles within mitochondria (**D**), the cytosolic amount of PARKIN (**E**), and the ratio of mitochondrial to cytosolic PARKIN particles (**F**). Values reported in the graph correspond to the mean ± SD. Mitochondria were counted in 50 cells per group. * *p* ≤ 0.05 compared with controls. ** *p* ≤ 0.05 compared with controls and 21 d. *** *p* ≤ 0.05 compared with other groups. Scale Bar = 0.5 μm, Asterisk (*) = mitochondria, ER = endoplasmic reticulum.

Similar to LC3, though more evident, is the polarization of ULK1 immuno-gold within mitochondria (representative Figure 7A,B and graphs of Figure 7C,D). Indeed, the increase of ULK1 within mitochondria surpasses, at large, the increase of ULK1 within cytosol (as counted in the graphs of Figure 7E,F). Remarkably, such a polarization is evident also for AMBRA1 (representative pictures at Figure 8A,B). In fact, the stoichiometric count of AMBRA1-stained mitochondria (graph of Figure 8C) and the amount of the protein AMBRA1 within mitochondria (Figure 8D), indicate a selective increase within mitochondria compared with a slight decrease in the cytosol (Figure 8E), which leads to the highest mitochondria/cytosol ratio for AMBRA1 compared with other mitophagy protein (Figure 8F). This is consistent with the powerful effects induced by mitophagy stimulation through the interaction of AMBRA1 with LC3 [37,38,39]. In the context of targeting mitochondrial removal, the placement of PARKIN within mitochondria is key to form a complex with PINK1 which triggers mitophagy [39,40]. In the present study, it is shown that, expression of the PARKIN protein is suddenly increased by rapamycin (Representative Figure 9A,B), thus focusing its effects at the mitochondrial level (Figure 9C,D). This occurs despite a generalized increase of PARKIN in the cytosol (Figure 9E), though occurring to a lesser extent, as shown by the mitochondrial vs. cytosolic ratio of PARKIN immuno-gold (Figure 9F). When comparing the increase of PARKIN compared with ULK1 and AMBRA1 it is remarkable that mitochondrial polarization of PARKIN occurs maximally shortly after the rapamycin challenge.

**Figure 10:**
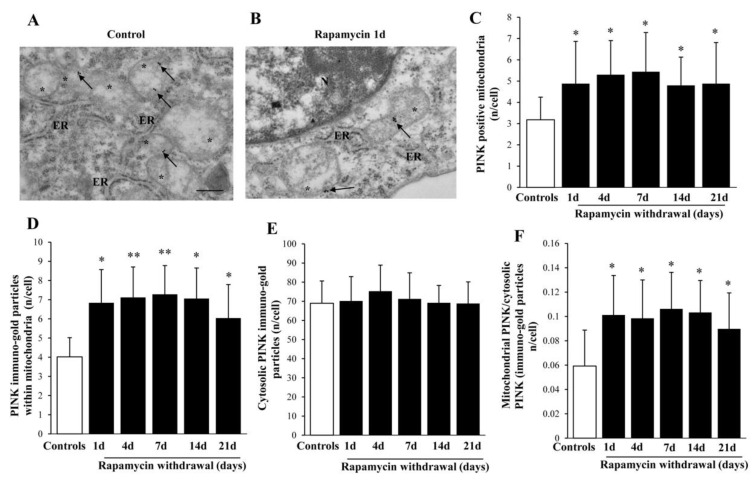
Representative immuno-gold and polarization graphs for PINK1. Representative TEM micrograph showing PINK1-positive mitochondria from control (**A**) and following 1 d rapamycin withdrawal (**B**). Arrows point to PINK1 immuno-gold particles within mitochondria. Graphs report the PINK1-positive mitochondria (**C**), PINK1 immuno-gold particles within mitochondria (**D**), the cytosolic amount of PINK1 (**E**), and the ratio of mitochondrial to cytosolic PINK1 particles (**F**). Values reported in the graph correspond to the mean ± SD. Mitochondria were counted in 50 cells per group. * *p* ≤ 0.05 compared with controls. ** *p* ≤ 0.05 compared with controls and 21 d. Scale Bar = 0.5 μm, Asterisk (*) = mitochondria, ER = endoplasmic reticulum, N = nucleus.

This suggests that PARKIN plays a key role in initiating the mitophagy process despite persisting for a very long time (at least 21 days). Such an effect is mirrored by its molecular partner PINK1 (representative pictures at Figure 10A,B), which persists elevated for at least 21 d, albeit, selectively at mitochondrial level (graph of Figure 10C,D). In fact, in the cytosol there is noticeable increase in PINK1 at any time interval (Figure 10E), with a marked ratio between mitochondria and cytosol (Figure 10F). Such an effect resembles what described for AMBRA1, which is consistent with the canonical pathway of mitophagy. This happens though PINK1 phosphorylation at mitochondrial level, which makes it able to recruit and interact with PARKIN to promote mitochondrial removal [41].

### 2.9. Within Mitochondria, Rapamycin Increases the Fission Proteins FIS1 and DRP1, While the Fusion Protein OPA1 Is not Changed

**Figure 11:**
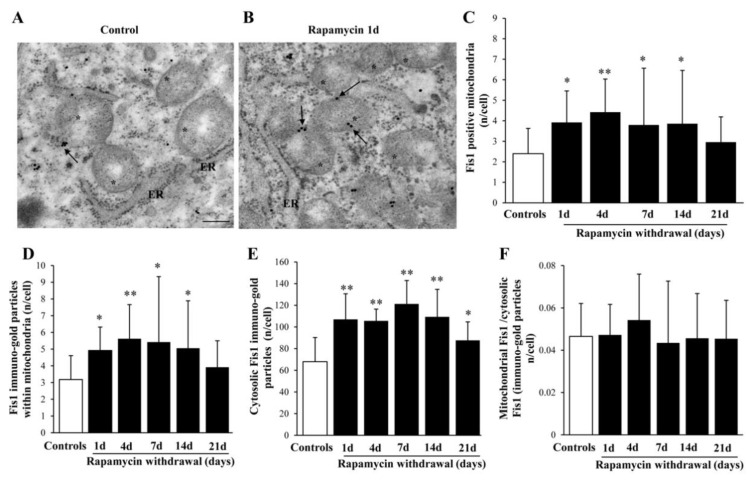
Representative immuno-gold and polarization graphs for the mitochondrial fission marker FIS1. Representative TEM micrograph showing FIS1-positive mitochondria from control (**A**) and following 1 d rapamycin withdrawal (**B**). Arrows point to FIS1 immuno-gold particles within mitochondria. Graphs report the FIS1-positive mitochondria (**C**), FIS1 immuno-gold particles within mitochondria (**D**), the cytosolic amount of FIS1 (**E**), and the ratio of mitochondrial to cytosolic FIS1 particles (**F**). Values reported in the graph correspond to the mean ± SD. Mitochondria were counted in 50 cells per group. * *p* ≤ 0.05 compared with controls. ** *p* ≤ 0.05 compared with controls and 21 d. Scale Bar = 0.5 μm, Asterisk (*) = mitochondria, ER = endoplasmic reticulum.

**Figure 12:**
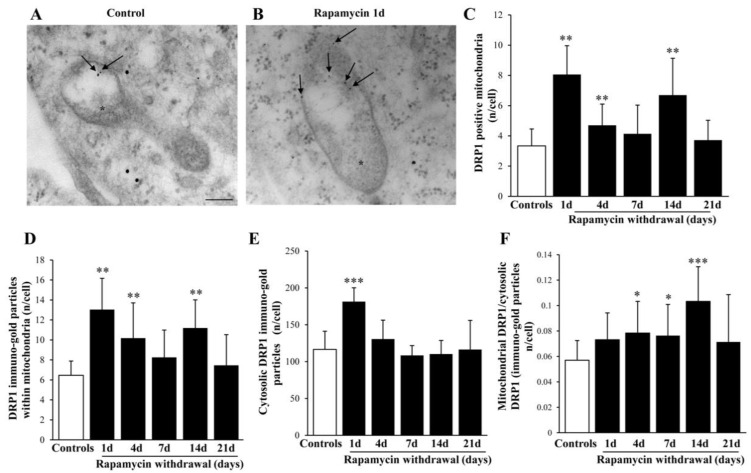
Representative immuno-gold and polarization graphs for the mitochondrial fission marker DRP1. Representative TEM micrograph showing DRP1-positive mitochondria from control (**A**) and following 1 d rapamycin withdrawal (**B**). Arrows point to DIRP1 immuno-gold particles within mitochondria. Graphs report the DRP1-positive mitochondria (**C**), DRP1 immuno-gold particles within mitochondria (**D**), the cytosolic amount of DRP1 (**E**), and the ratio of mitochondrial to cytosolic DRP1 particles (**F**). Values reported in the graph correspond to the mean ± SD. Mitochondria were counted in 50 cells per group. * *p* ≤ 0.05 compared with controls. ** *p* ≤ 0.05 compared with controls and 21 d. *** *p* ≤ 0.05 compared with other groups. Scale Bar = 0.5 μm, Asterisk (*) = mitochondria.

**Figure 13:**
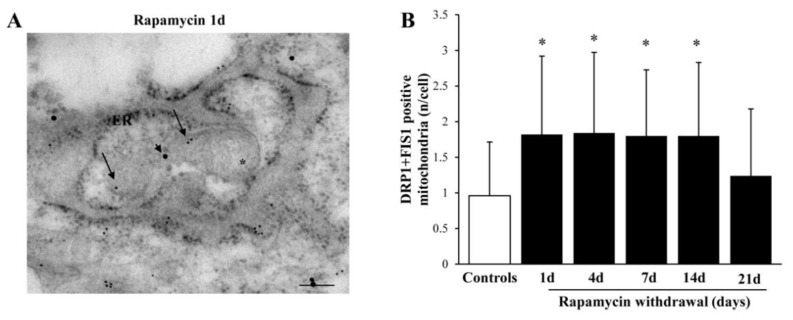
Rapamycin increases the combination of fission markers FIS1 and DRP1. (**A**) Representative mitochondrion double stained for FIS1 and DRP1 immuno-gold particles (20 nM, arrowhead, and 10 nM, arrows respectively) following 1 d rapamycin withdrawal. Graph (**B**) reports FIS1+DRP1-positive mitochondria. Values reported in the graph correspond to the mean ± SD. Mitochondria were counted in 50 cells per group. * *p* ≤ 0.05 compared with controls. Scale Bar = 0.25 μm, Asterisk (*) = mitochondria, ER = endoplasmic reticulum.

**Figure 14:**
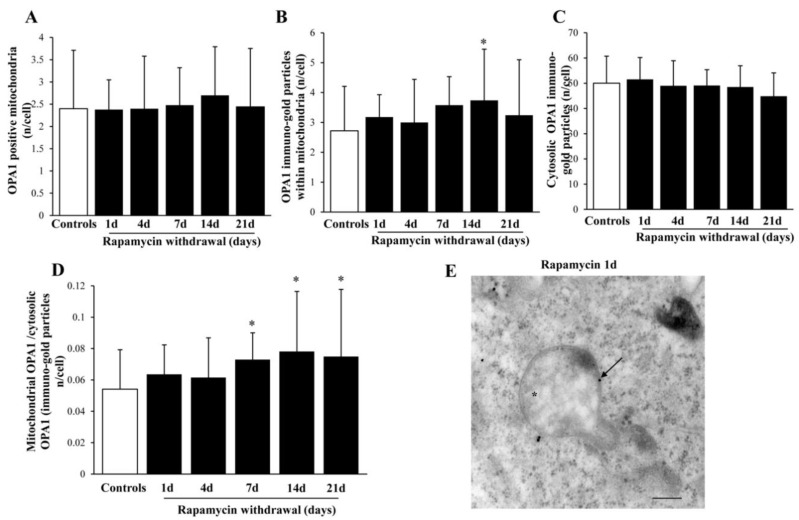
Representative immuno-gold and polarization graphs for the mitochondrial fission marker OPA1. Graphs report the OPA1-positive mitochondria (**A**), OPA1 immuno-gold particles within mitochondria (**B**), the cytosolic amount of OPA1 (**C**), and the ratio of mitochondrial to cytosolic OPA1 particles (**D**). Representative TEM micrograph showing OPA-positive mitochondria from control (**E**). Arrows point to OPA1 immuno-gold particles within mitochondria. Values reported in the graph correspond to the mean ± SD. Mitochondria were counted in 50 cells per group. * *p* ≤ 0.05 compared with controls. Scale Bar = 0.3 μm, Asterisk (*) = mitochondria.

The fission marker FIS1 increases at all time intervals under analysis (Figure 11A,B) persistingly elevated at 14 d of rapamycin withdrawal. Within mitochondria FIS1 undergoes an increase, which also occurs in the cytosol (Figure 11C,F). Similarly, the fission antigen DRP1 augments following rapamycin (representative Figure 12A and Figure 13B). However, differing from FIS1, DRP1 increases more focally within mitochondria (Figure 12C,D), compared with cytosol (Figure 12E). When immuno-gold for FIS1 and DRP1 were combined, the co-staining at mitochondrial level, was remarkable and persisted for 21 d. This combined staining provides information on the interaction of FIS1 and DRP1. This is needed to promote mitochondrial fission as shown in a representative picture of a mitochondrial fission of Figure 13A and reported in graph of Figure 13B. In contrast, OPA1 level were neither altered nor significantly compartmentalized at early or mean time intervals (graphs of Figure 14A–C). Only a late increase in the ratio between mitochondrial and cytosolic OPA1 was detected (graph of Figure 14D). This is likely to depend on the switch in the fission/fusion cycle, where fusion rebounds after a binging of fission [42,43,44], when abundant mitochondria persist for weeks in the cell and may need to merge as shown in representative picture showing mitochondrial fusion in Figure 14E.

## 3. Discussion

The present research study quantifies some facets of mitochondrial status in baseline condition and following rapamycin within GBM cells. These cells in baseline conditions feature a marked suppression of autophagy due to mTOR upregulation [5,10].

Despite occurrence of a high number of altered mitochondria are observed in baseline conditions, these cannot be efficiently removed due to an ongoing defect in mitophagy as reported by Fan et al. [45]. Therefore, altered mitochondria accumulate in the cell. Alternatively, other pathways are reported to contribute to the clearance of altered mitochondria. For instance, in patients-derived GBM stem cells, altered mitochondria are transferred to neighboring cells through tunneling nanotubes in the attempt to get reed of dysfunctional organelles [46]. Such a cell-to-cell alternative mitochondrial clearance also occurs between stromal and GBM cells [47].

Evidence is provided here showing that, exposure for 24 h to 10 nM rapamycin steady increases mitochondrial number at least for 21 d following rapamycin withdrawal.

Similarly, the amount of altered mitochondria diminishes steadily for 21 d. It is remarkable that both effects persist for a similar amount of time, which suggests that analogous phenomena were causing both increased mitochondrial number and improved mitochondrial morphology. Remarkably, mitochondrial repair and removal quantified at TEM is consistent with mTOR dependent activation of mitochondrial fission and mitophagy genes.

Genes specifically involved in mitochondrial fission and mitophagy, by acting in combination concretely remove altered mitochondria and increase the amount of healthy organelles. The significance of an increase in those genes promoting fission should be further considered in the light of a reciprocal and mutual influence. In fact, while fission-related genes also inhibit fusion [42,43,44,48]; the fusion gene OPA1 inhibits fission [42,44,48]. Again, fission related genes may also foster another seminal step in mitochondrial dynamics which concerns mitophagy [37,38,39,40,41,42,44,49,50,51,52,53,54].

In line with gene expression, we could document an increase of protein detected stoichiometrically, in situ, within mitochondria and scattered in the cytosol. Almost all these proteins were markedly polarized towards mitochondria by rapamycin. This is the case also for LC3, which, despite its vacuolar compartmentalization, is similarly concentrated at mitochondrial level. Analogous findings were obtained when the specific mitophagy proteins ULK1, AMBRA1, PARKIN, and PINK1, which own a significant role in mitophagy. The same happened for fission-related proteins FIS1 and DRP1.

The inhibition of mTOR by acting on these steps of mitochondrial dynamics improves the mitochondrial status in GBM cells contributing to the increase in well-structured mitochondria, which compensate for GBM-specific mitochondrial defect. Thus, when mitophagy is activated, mitochondrial dynamics is synergistically modified [10,55,56,57] by increasing mitochondrial fission.

In turn, in these experimental conditions a pronounced mitochondrial biogenesis takes place as recently shown under rapamycin administration [10]. This indicates the occurrence of mTOR-dependent cascade, which operates according to an orchestrated pattern, where mitophagy and mitochondrial biogenesis coordinate each other [57,58]. Remarkably, the effects described here were long-lasting with at least three weeks of rapamycin withdrawal. This suggests a signaling which brings to mitochondrial plasticity under the control of mTORC1 activity.

When describing cell pathology affecting GBM, an autophagy defect and a mitochondrial dysfunction are generally reported as independent phenomena, while as shown here, both may stem from the same molecular events. It is remarkable that, compartmental polarization, which was previously supposed to solely recruit the autophagy vacuolar compartment, extends even to a higher extent at the level of mitochondria. In the present study, we specifically detected an increase in those mitochondria characterized by small size, remarkable architecture, and strong electron density. This is related to an increased mitophagy and mitochondrial fission, which add on mitochondrial biogenesis previously counted in these experimental conditions. It is remarkable that a number of molecules analyzed in this study as part of specific pathways involved in mitochondrial dynamics are modified persistently and harmonically following rapamycin administration. The persistency of rapamycin-induced gene expression translates into persistent protein and organelle changes, which appears as a strong evidence for plastic phenomena.

The limits of the present study rely on a single cell line being analyzed. Previous findings obtained in primary GBM cell cultures from various patients [5] as well as in multiple cell lines from different sources (A172, U251) [6,10] confirm that, in GBM an mTOR–dependent pathway is overactive. In fact, rapamycin acts in these variable settings according to the very same dose–response curve. Due to the tightened relationship between autophagy and mitophagy, and the mTOR-dependency of both phenomena, it is likely that these findings may have general implications in GBM.

## 4. Materials and Methods

### 4.1. Experimental Design

Experiments were carried out in Uppsala derived human cell line (U87MG) obtained from Cell Bank (IRCCS San Martino-Institute, Genova, Italy). U87MG cells were maintained in DMEM growth medium (Sigma-Aldrich, Saint Louis, MO, USA) containing 10% Fetal Bovine Serum (FBS, Sigma-Aldrich), 1% of MEM Non-Essential Amino-Acid (MEM-NEAA, Sigma-Aldrich), penicillin, and streptomycin (50 IU/mL and 100 μg, respectively, Sigma-Aldrich) and kept at 37 °C in a humidified atmosphere containing 5% CO_2_.

We applied an experimental design where gene expression and specific ultrastructural morphometry were evaluated following a short administration of rapamycin. Experiments were designed to measure the persistency of genetic and ultrastructural effects produced by rapamycin on mitochondrial morphometry, and genes regulating mitophagy, fission, and fusion. This is measured concomitantly with protein stoichiometry within whole cell and in situ, within mitochondria, where the proteins coded by these genes including their sub-cellular placement were quantified. Therefore, a time course was carried out at 1 d, 4 d, 7 d, 14 d, and 21 d following rapamycin withdrawal. This time course is based on previous evidence of long-lasting alterations induced by a short exposure to rapamycin [5,6].

Rapamycin was administered for a short time interval (24 h). Such a short time was selected in pilot studies by using either MitoTracker-Red and plain TEM to quantify mitochondrial number and mitochondrial alteration, as reported in Figures S1–S5. The doses of rapamycin to be tested were selected in order to include its therapeutic range (3–15 nM) [59]. In fact, rapamycin was administered in pilot experiments at three different doses (1 nM, 10 nM, 100 nM). The dose of 10 nM was selected for all the experiments reported in the body of the manuscript for ultrastructural morphometry. Nonetheless, in gene expression experiments we always report two doses of rapamycin, which sometime provides discrepant effects (the 10 nM dose and a higher dose of 100 nM based on the experiments by Supko and Malspeis [60]). These experimental conditions concerning timing and dosing of early rapamycin exposure were kept constant in all further experimental steps designed to assess mitochondrial gene expression and dynamics (mitophagy, fission, and fusion) at various time interval following withdrawal of rapamycin administered.

After rapamycin exposure, the cell culture was washed to remove rapamycin from the cells, which were further re-washed (every three days culture medium was removed and replaced with fresh medium, to keep them alive for long time intervals required by this protocol). Dilutions of rapamycin were prepared from a stock solution of 1 mM rapamycin dissolved in 1.41 M DMSO, which was further solved in culture medium when administered to the cell culture. In this way, the final concentration of DMSO was 0.01%. Control cells were grown in the same culture medium containing 0.01% DMSO for the same time intervals with the same washing procedure.

### 4.2. RNA Extraction

TRIzol Reagent (Thermo Fisher Scientific, Waltham, MA, USA) was used to isolate total RNA from cell culture. Nanodrop 2000 (Thermo Fisher Scientific) was used to determine the concentration and purity of RNA samples. Total RNA (100 ng) was reversely transcribed (RT) with SuperScript^®^ VILOTM (Thermo Fisher Scientific), according to the manufacturer’s instructions.

### 4.3. qReal-Time-PCR

Amplification and detection were performed on a CFX ConnectTM Real Time System (Bio-Rad, Hercules, CA, USA). PCR mix including 10 μL SYBR Green PCR Master (Applied Biosystems, Foster City, CA, USA), 0.5 μM of each primer and 0.8 μL of RT reaction mix, was amplified as follows: 50 °C for 1 min, 95 °C for 10 min followed by 40 cycles of 95 °C for 30 s, 54 °C for 1 m. The primers have been designed using GenBank (http://www.ncbi.nlm.nih.gov/, Table 1, accessed on 7 November 2018).

Positive controls (DNA), negative control (distilled water), and RT-negative controls (total RNA sample) were included in each run.

The relative quantification was calculated using comparative Ct method (also known as the ΔΔCT method) [61,62], with beta-globin and beta-actin which were selected as internal reference. Ct values correspond to mean values of each PCR performed in triplicate. Gene expression was confirmed in two independent experiments using both beta-globin and beta-actin as internal reference. Real time PCR was carried out to measure different steps of mitochondrial dynamics by selecting the following genes: (i) Mitophagy (*VPS34; ULK1; AMBRA1*); (ii) fission (*FIS1; DRP1*); and (iii) fusion (*OPA1*). In addition, specific genes, which are key to trigger mitophagy (*PINK1*), or to shuttle mitochondria to the phagophore (*PARKIN*), were quantified. The latter may be considered as a single functional unit (defined as the PINK1–PARKIN complex), which is activated when mitochondrial clearance is required [39,41].

Thus, after a short challenge with rapamycin, the experimental design proceeded at 1 d, 4 d, 7 d, 14 d, and 21 d of rapamycin withdrawal.

### 4.4. MitoTracker-Red

In order to check whether the number of healthy mitochondria in living cells undergo an increase under the effects of rapamycin compared with GBM cells in baseline conditions, we carried out fluorescence for MitoTracker-Red dye [34]. Briefly, 5 × 10^2^ GBM cells were grown in 24 well plates containing 1 mL/well of culture medium. At the end of each experiment, the medium was removed and cells were incubated with a solution of MTR 250 nM in a serum free culture medium for 45 min, at 37 °C, and 5% CO_2_. At the end of incubation, MTR solution was removed and fresh pre-warmed medium was added. Stained cells were analyzed at fluorescence microscopy (Leica Microsystems); optical density was calculated using ImageJ software.

### 4.5. Transmission Electron Microscopy (TEM)

For TEM, U87MG cells (10^6^) were seeded into 10 mm diameter dishes with 5 mL of culture medium. After removing culture medium, a fixing solution (2.0% paraformaldehyde/0.1% glutaraldehyde, both dissolved in 0.1 M PBS pH 7.4) was added to the cell culture for 90 min at 4 °C. Cells were gently scraped from the plate, collected into vials, and centrifuged at 10,000 rpm for 10 min to deposit a cell pellet. After removing supernatant, this pellet was re-suspended in PBS and centrifuged again at 8000 rpm. After removing supernatant, the second pellet which was free of aldehydes remnants, was post-fixed in 1% osmium tetroxide (OsO_4_) for 1 h at 4 °C. After washing in PBS the post-fixed pellet was dehydrated in a gradient of ethanol solutions 50%, 70%, 90%, and 95%, each for 5 min, to reach 100% ethanol for 60 min. This was followed by adding a solution of propylene oxide for 15 min to produce an interface between the samples and the epoxy resin, which is more compatible compared with alcohol. In fact, when propylene oxide was removed the pellet was progressively embedded, at first in a 50% solution of epon-araldite in propylene oxide, overnight. Finally, the pellet was embedded in pure epon-araldite for 72 h, at 60 °C to improve resin polymerization. Following removal from the oven, after a short interval to cool the embedded sample, this was a firm material, ready for cutting. Thus, ultrathin sections could be firmly and homogeneously cut at ultra-microtome (Leica Microsystems) and either plain TEM or post-embedding immuno-electron microscopy were carried out. Ultrathin sections were observed at Jeol JEM SX100 electron-microscopy (Jeol, Tokyo, Japan) at an acceleration voltage of 80 kV.

#### 4.5.1. Post-Embedding Immuno-Electron Microscopy

Post-embedding immuno-electron microscopy is a procedure, which requires optimal fixing of the tissue and its embedding. In previous studies [39,63,64,65] we validated the use of OsO_4_ and epoxy resin to preserve ultrastructural morphometry [39,63,64,65,66]. In fact, a combination of aldehydes, OsO_4_, and epoxy resin allows a minimal epitope covering, while preserving sub-cellular architecture. In particular, OsO_4_ binds to lipid membranes, making them well marked compared with surrounding material. This allows specific detection of each organelle contour and, acting as a contrast enhancer magnifies the subcellular trim. Moreover, epoxy resin better preserves cell architecture compared with acrylic resin.

Post-embedding immuno-electron microscopy was carried out to test different antibodies in ultrathin sections. We used gold-conjugated secondary antibodies, which allow stoichiometric detection and localization of the antigens within sub-cellular structures [67]. Ultrathin sections were collected on nickel grids and processed for protein detection, as detailed in Table 2. The oxidizing agent sodium metaperiodate (NaIO_4_) was used to partially remove OsO_4_ as much as needed to unmask antigens [68]. The sodium metaperiodate attacks the hydrophobic alkane side-chains of epoxy resin thus making sections more hydrophilic, which allows an intimate contact between immuno-gold-conjugated antibodies and section surface antigens [63,64,65]. Nickel grids were incubated with aqueous saturated NaIO_4_ solution for 30 min, at 21 °C.

Here, we report the rough list of primary antibodies used: (i) LC3, (Abcam, Cambridge, UK), (ii) VPS34 (Thermo Fisher Scientific), (iii) ULK1 (Cell Signaling Thechnology, Danvers, MA, USA), (iv) AMBRA1 (Abcam), (v) PARKIN (Millipore, Burlington, MA, USA), (vi) PINK1 (Abcam), (vii) FIS1 (GeneTex, Irvine, CA, USA), (viii) DRP1 (Abcam). (ix) OPA1 (Abcam).

After washing in PBS, grids were incubated on drops of blocking buffer (10% goat serum and 0.2% saponin in PBS) for 20 min, at 21 °C, and then, with a single primary antibody (diluted 1:20, in PBS containing 1% goat serum and 0.2% saponin) or with two antibodies in order to detect co-localization (FIS1+DRP1 both diluted 1:20) in a humidified chamber overnight at 4 °C.

After washing in cold PBS, ultrathin sections were incubated on drops of blocking buffer containing gold-conjugated secondary antibodies (10 nm gold particles; 20 nm gold particles, BB International, Treviso, Italy), diluted (1:20) for 1 h at 21 °C. Then, grids were incubated with 1% glutaraldehyde for 3 min, and they were washed in distilled water to remove salt traces and prevent uranyl-acetate precipitation. Grids were counterstained with a saturated solution in distilled water of uranyl acetate and lead citrate to be ready for electron microscopy. To control for method some sections were incubated with secondary antibody only.

#### 4.5.2. Ultra-Structural Analysis of Mitochondria

Grids containing non-serial ultrathin sections (90 nm thick) were examined at 6000× magnification to count both total number and altered number of mitochondria per cell. The number of immuno-gold particles was counted at 8000×. Several grids were observed in order to count a number of at least 50 cells per each experimental group. Starting from a grid square corner, the whole sectioned pellet embedded within that grid square was scanned in equally spaced parallel sweeps across the specimens. The counts were repeated at least twice by two blind observers. Mitochondria were easily identified at TEM for their shape and structure consisting in a typical double-membrane limiting an inter-membrane (“inter-mitochondrial”) space and an area internal to the inner membrane, where a homogeneous matrix is interrupted quite regularly by intermingled crests (cristae), in a sort of labyrinth system. Although the general morphology of the mitochondria is standardized at ultrastructural level, many variations may be noticed at high magnification within the context of specific cell metabolism and cytopathology [10,69]. All these variants can be appreciated concomitantly, when working in GBM cells. Nonetheless, under the effects of rapamycin there is an evident shift towards a specific morphology, which will be reported in the results section. In fact, when control cells possess a defect in mitochondrial removal and fission and organelles tend to merge and enlarge, the effects of rapamycin promote the opposite phenotype, as we shall see.

### 4.6. Statistics

For MitoTracker-Red, stained cells were analyzed at fluorescence microscopy (Leica Microsystems); optical density was calculated using ImageJ software. Values are expressed as the percentage of the fluorescent densitometry of each sample with respect to the controls (N = 50). Inferential statistics to compare groups was carried out by using One-way analysis of variance, ANOVA, with Scheffè’s post-hoc analysis (H_0_, null hypothesis, was rejected for *p* ≤ 0.05).

For qReal Time-PCR experiments, statistical analyses using one-way analysis of variance (ANOVA) followed by Bonferroni test, using GraphPad Prism version 6.0 has been used to test differences in gene expression in baseline conditions and under the effects of rapamycin (*p* ≤ 0.05).

For ultrastructural morphometry, the number of total and altered mitochondria per cell were quantified along as the number of immuno-gold particles both in the cytosol and within mitochondria and the cytosolic/mitochondrial ratio for the following antigens: LC3, VPS34, ULK1; AMBRA1, PARKIN, PINK1, FIS1, DRP1, OPA1. Data are the mean or the mean percentage±SD per cell (N = 50 cells per group). Inferential statistics to compare groups was carried out by using One-way analysis of variance, ANOVA, with Scheffè’s post-hoc analysis (H_0_, null hypothesis, was rejected for *p* ≤ 0.05).

## Figures and Tables

**Table 1:** Primers used for quantitative Real Time PCR.

Target Gene	Target Sequence Number	Primer Sequences
*DRP1*	NM_001330380.1	FW 5′-AGCTGCTCAGTATCAGTCTC-3′ RW 5′-GGCAGTCAAAATGTCAATAGT-3′
*FIS1*	NM_016068.3	FW 5′-TCAGTCTGAGAAGGCAGCA-3′ RW 5′-CGCTGTTCCTCCTTGCT-3′
*OPA1*	NM_130837.2	FW 5′-TTCAGTATCAGCAAAGCT-3′ RW 5′-GAGGGTCCATTTGACTGAC-3′
*ULK1*	NM_003565.3	FW 5′-CAGACGACTTCGTCATGGTC-3′ RW 5′-AGCTCCCACTGCACATCAG-3′
*VPS34*	NM_002647.4	FW 5′-GGGGAAGCAGAGAAGTTTCA-3′ RW 5′-TCTTCCCTTCCAAGCTTCCT-3′
*AMBRA1*	NM_001367468.1	FW 5′-GAGCACCCAATTTACCCAGA-3′ RW 5′-GATCATCCTCTGGGCGTAGTA-3′
*PARKIN*	NM_004562.3	FW 5′-TGAGAAGCTGGATTACCATC-3′ RW 5′-TTGAGAGTGACACAGATGACCT-3′
*PINK1*	NM_032409.3	FW 5′-GGCTTTCGGCTGGAGGAGTA-3′ RW 5′-GCTCGCTGGGACCAGCTCC-3′
*β–Actin*	NM_001101.3	FW 5′-GTGCGTGACATTAAGGAG-3′ RW 5′-GCCAGACAGCACTGTGT-3′
*β-Globin*	NM_000518.4	FW 5′-CTAAGGTGAAGGCTCATG-3′ RW 5′-GATAGGCAGCCTGCACT-3

**Table 2:** Sources and identification of each antibody used in the study.

Antibodies	Distributors	Cod. N°	RRID
Rabbit anti-LC3	Abcam	ab128025	AB_11143008
Rabbit anti-FIS1	GeneTex	GTX111010	AB_10731288
Mouse anti-DRP1	Abcam	ab56788	AB_941306
Rabbit anti-OPA1	Abcam	ab90857	AB_2050139
Mouse anti-PINK1	Abcam	ab186303	AB_2827698
Rabbit anti-PARKIN	Millipore	AB5112	AB_2283497
Rabbit anti-ULK1	Cell Signaling Technology	D8H5	AB_11178668
Rabbit anti-AMBRA1	Abcam	ab 59141	AB_941614
Rabbit anti-Vps34	Thermo Fisher Scientific	PA1-46456	AB_2283904
EM Goat anti-Rabbit IgG, gold conjugated antibody	BBInternational	EM.GAR 20	AB_1769136
EM Goat anti-Rabbit IgG, gold conjugate antibody	BBInternational	EM.GAR 10	AB_1769128

## Supplementary Materials

The following are available online at https://www.mdpi.com/article/10.3390/ijms22105379/s1.
