# Supplementary material for: Rapamycin Ameliorates Defects in Mitochondrial Fission and Mitophagy in Glioblastoma Cells
Source: Int J Mol Sci. 2021 May 20;22(10):5379. doi: 10.3390/ijms22105379 (PMC8161366; doi:10.3390/ijms22105379)
Supplement: Supplementary file 1 [file ijms-22-05379-s001.zip › ijms-1213319-supplementary.pdf]

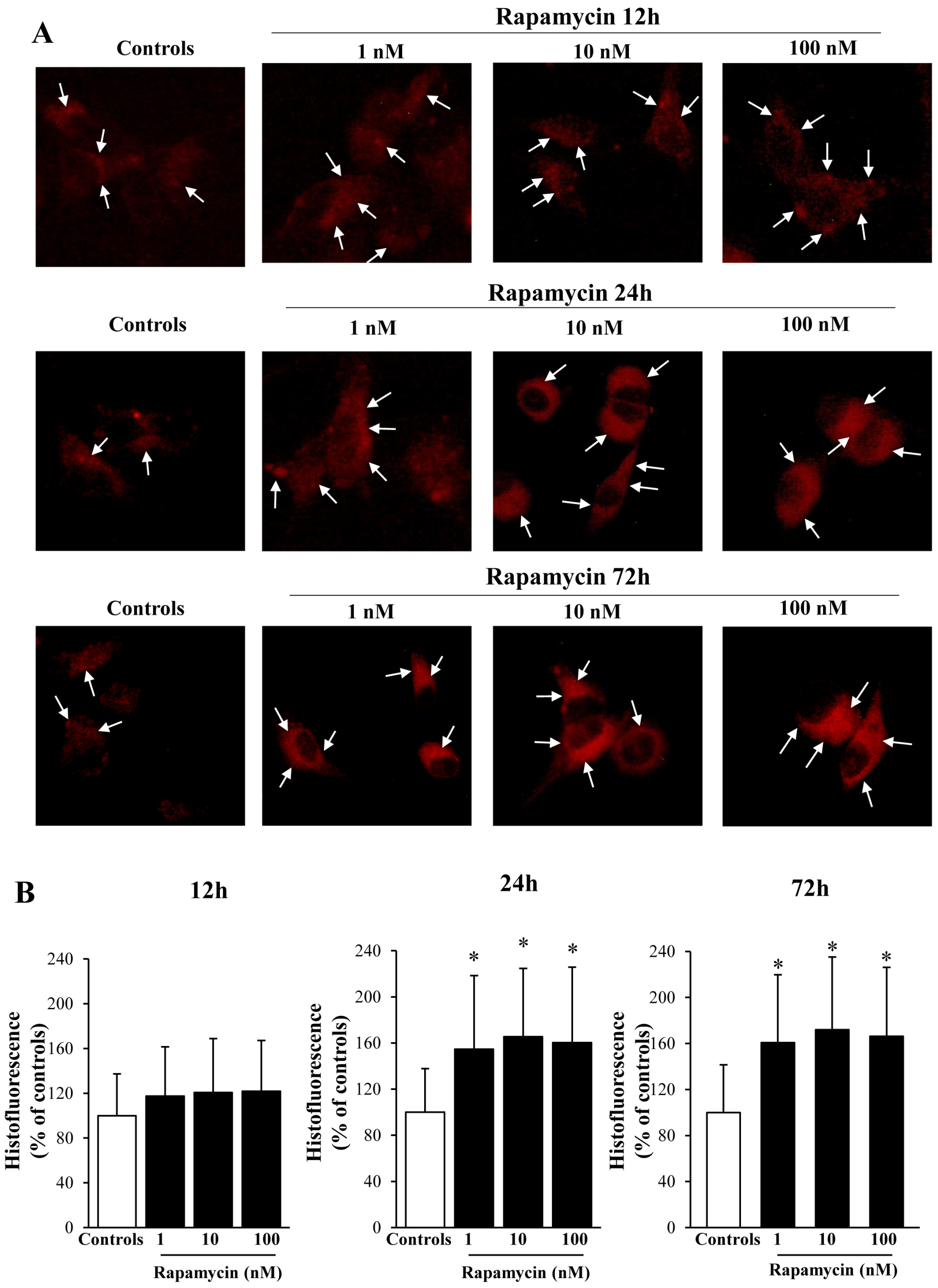

Legend to Figure S1. (**A**) Representative pictures stained with MitoTracker-Red showing healthy mitochondria (arrows) from control and rapamycin (1 nM, 10 nM, 100 nM) continuous exposure for (12h, 24h, 72h). (**B**) Graphs showing the percentage of MitoTracker-Red histofluorescence compared with control (histofluorescence, % of control) at 12h (left graph); 24h (middle graph); 72h (right graph) of continuous exposure to various doses of rapamycin (1 nM, 10 nM, 100 nM).

Counts represent the mean±SD from N=50 cells per group.

*P ≤ 0 05 compared with control. Scale Bar=20 μm.

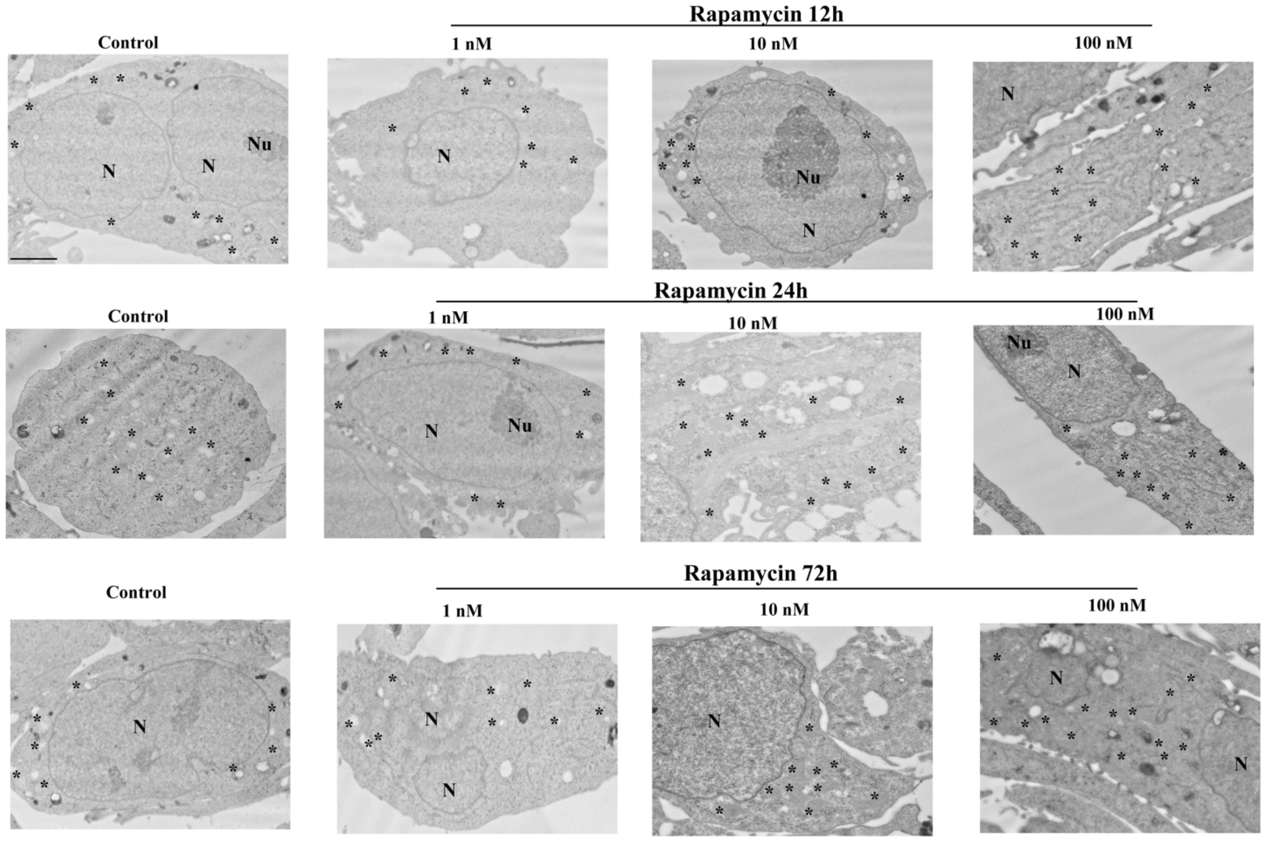

Legend to Figure S2. Representative TEM micrographs (low magnification) showing mitochondria (*) from control and rapamycin (1 nM, 10 nM, 100 nM) continuous exposure (12h, 24h, 72h). Scale Bar=3 μm, N=nucleus, Nu=nucleolus.

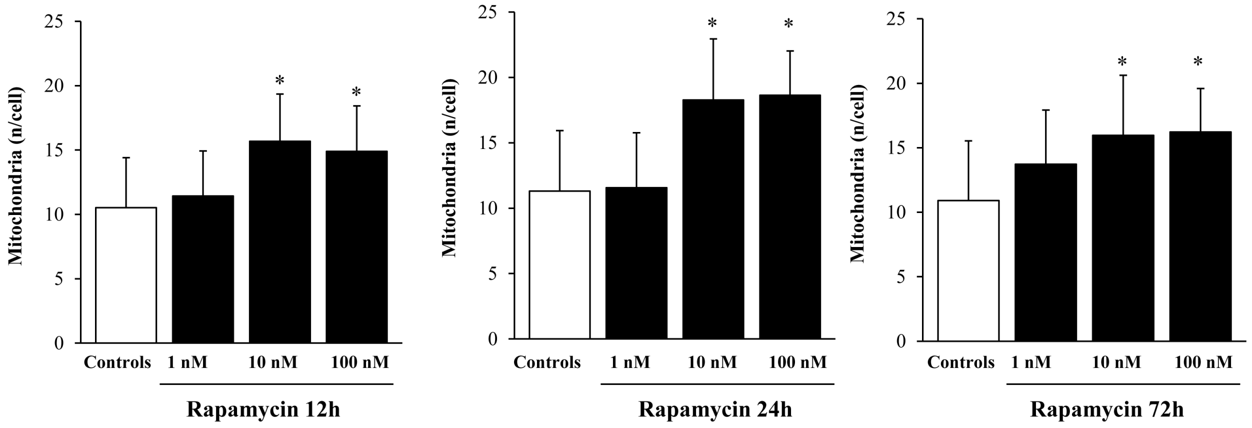

Legend to Figure S3. Graphs showing the number of mitochondria per cell at 12h (left graph); 24h (middle graph); 72h (right graph) of continuous exposure to various doses of rapamycin (1 nM, 10 nM, 100 nM).

Counts represent the mean±SD from N=50 cells per group.

*P ≤ 0.05 compared with control.

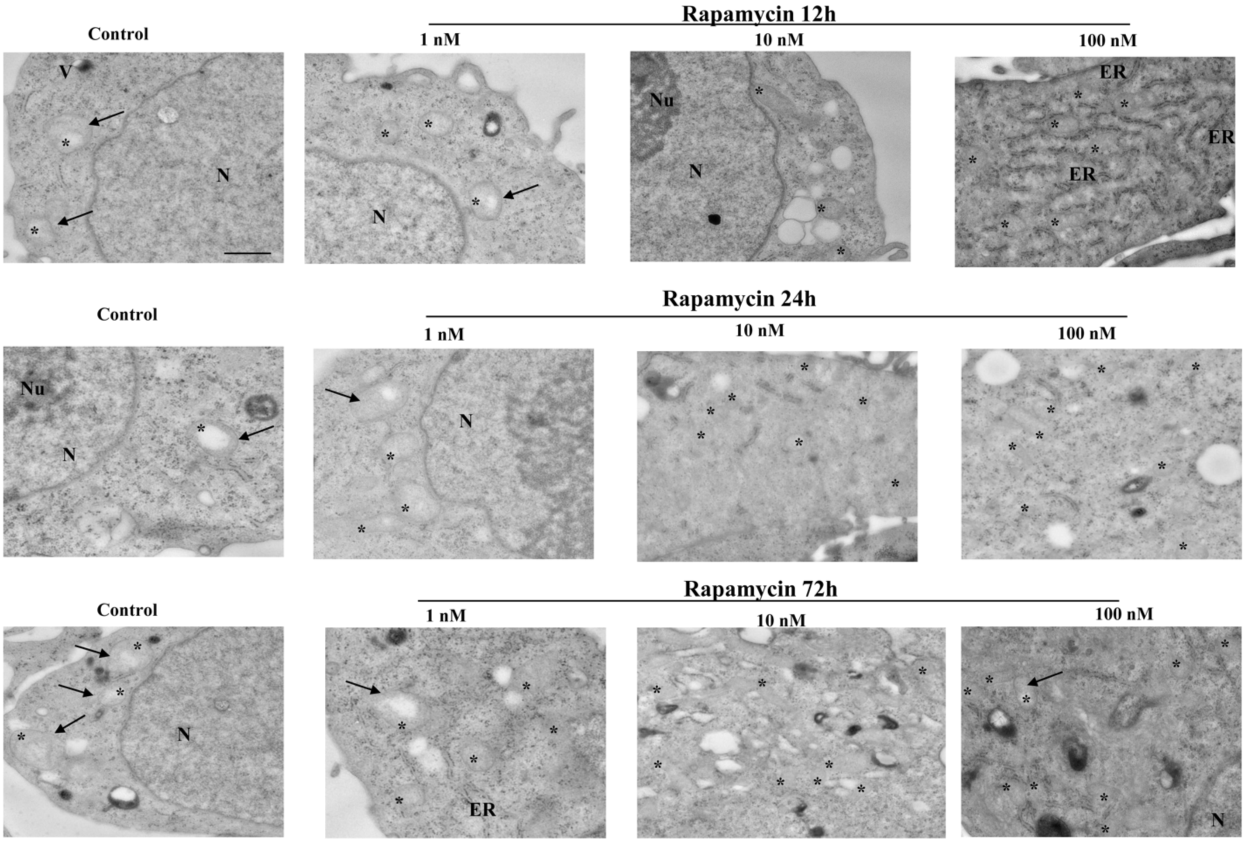

Legend to Figure S4. Representative TEM micrograph (high magnification) showing altered mitochondria (arrows) from control and rapamycin (1 nM, 10 nM, 100 nM) continuous exposure (12h, 24h, 72h).

Scale Bar=1 μm, Asterisk(*)=mitochondria, N=nucleus, Nu=nucleolus, ER=endoplasmic reticulum.

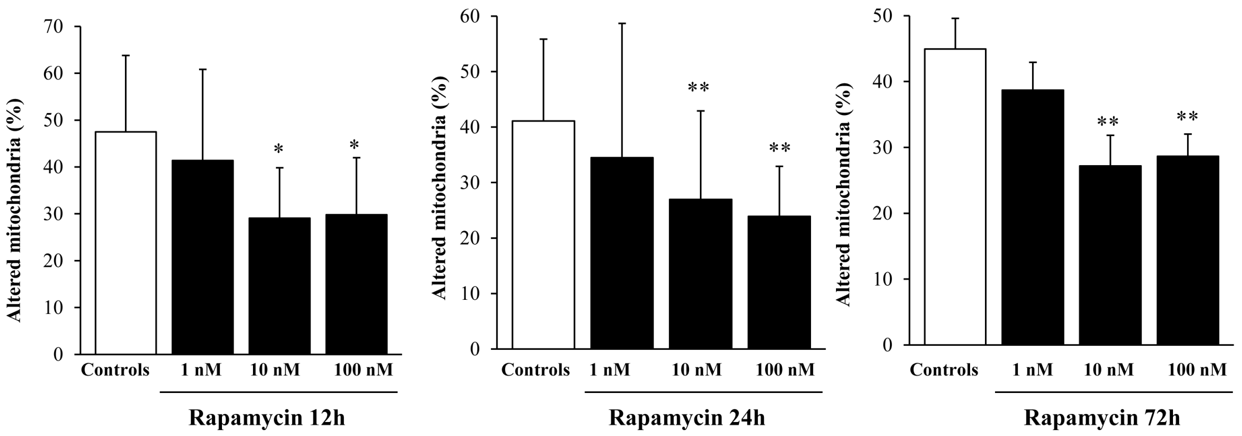

Legend to Figure S5. Graphs showing the percentage of altered mitochondria compared with control (altered mitochondria % of control) at 12h, 24h, 72h of continuous exposure to various doses of rapamycin (1 nM, 10 nM, 100 nM).

Counts represent the mean±SD from N=50 cells per group.

*P ≤ 0 05 compared with control.

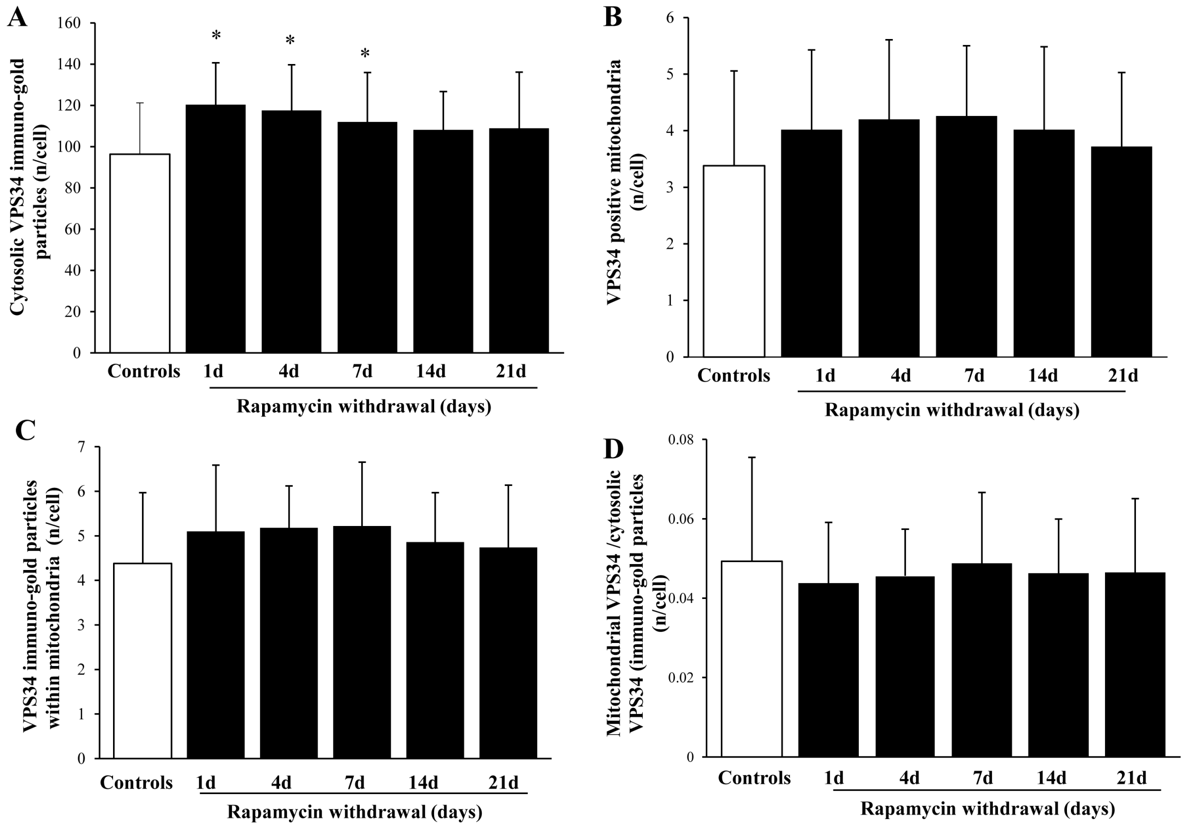

Legend to Figure S6. Representative immuno-gold and topographical morphometry graphs for early autophagy marker VPS34. Graphs report the cytosolic amount of VPS34 (**A**), the VPS34-positive mitochondria (**B**), the VPS34 immuno-gold particles within mitochondria (**C**) and the ratio of mitochondrial to cytosolic VPS34 immuno-gold particles (**D**).

Values reported in the graph correspond to the mean±SD from N=50 cells per group.

*P ≤ 0.05 compared with controls.
